# Impact of manganese and heme on biofilm formation of *Bacillus cereus* food isolates

**DOI:** 10.1371/journal.pone.0200958

**Published:** 2018-07-26

**Authors:** Mohammad Shakhawat Hussain, Minyeong Kwon, Deog-Hwan Oh

**Affiliations:** Department of Food Science and Biotechnology, College of Agriculture & Life Science, Kangwon National University, Chuncheon, Gangwon, South Korea; East Carolina University, UNITED STATES

## Abstract

The objective of this study was to determine the impact of manganese (Mn^2+^) and heme on the biofilm formation characteristics of six *B*. *cereus* food isolates and two reference strains (ATCC 10987 and ATCC 14579). The data obtained from the crystal violet assay revealed that addition of a combination of Mn^2+^ and heme to BHI growth medium induced *B*. *cereus* biofilm formation. However, the induction of biofilm formation was strictly strain-dependent. In all of the induced strains, the impact of Mn^2+^ was greater than that of heme. The impact of these two molecules on the phenotypic characteristics related to biofilm formation, such as cell density, sporulation and swarming ability, was determined in a selected food isolate (GIHE 72–5). Addition of Mn^2+^ and heme to BHI significantly (p < 0.05) increased the number of cells, which was correlated with the results of crystal violet assays as well as scanning electron microscopy (SEM) and confocal laser scanning microscopy (CLSM) analyses. In addition, induced biofilms showed higher numbers of spores and greater resistance to benzalkonium chloride. The swarming ability of *B*. *cereus* planktonic cells was increased in the presence of Mn^2+^ and heme in BHI. The expression levels of a number of selected genes, which are involved in mobility and extracellular polymeric substances (EPS) formation in *B*. *cereus*, were positively correlated with biofilm formation in the presence of Mn^2+^ and heme in BHI. These results further confirming the role of these molecules in swarming mobility and making matrix components related to *B*. *cereus* biofilm formation. These data indicate that signaling molecules present in the food environment might substantially trigger *B*. *cereus* biofilm formation, which could pose a threat to the food industry.

## Introduction

Bacterial biofilms are surface-attached multicellular communities enclosed by extracellular polymeric substances (EPS) [1]. Biofilm formation is significant in the food industry and in the environment as biofilms are very difficult to remove from attached surfaces and are resistant to disinfectants compared to their free-floating planktonic counterparts [2]. Bacterial biofilm formation can be influenced by a number of environmental factors, including nutrient composition, metabolites and co-factors present on attachment surfaces, and signaling molecules produced by bacteria [3–5]. Certain molecules induce a signaling pathway that leads to the synthesis of several EPS, and this plays a major role in promoting other biofilm-related phenotypes, including virulence and resistance to disinfectants [5–8].

*B*. *cereus* is a soil-dwelling, gram-positive, aerobic or facultative anaerobic bacterium. In addition to its presence in soil, it is ubiquitously found in other environments including raw and processed foods, milk, and water [9]. *B*. *cereus* is significant as it causes two types of food poisoning (emetic and diarrheal syndromes) and several local and systematic infections, including nosocomial infections, skin lesions and meningitis [10]. In addition, *B*. *cereus* is responsible for food spoilage, leading to huge economic losses in the food processing industry [11]. This bacterium is well known to attach to several biotic and abiotic surfaces and to form biofilms [12]. The phenotype of *B*. *cereus* biofilm formation is closely associated with high cell density, spore formation and resistance to disinfectants, making it a matter of serious public health concern and an issue with regard to food safety [3].

All organisms require manganese (Mn^2+^) and iron for their physiological needs and survival. The ability to sense Mn^2+^ and iron is particularly important for bacterial pathogenesis and colonization of specific environments [13]. Iron is the most abundant metal in the environment, but free ferrous iron (Fe^2+^) has extremely poor bioavailability. Heme, which constitutes an important source of iron, contains a single Fe^2+^ atom encircled by a tetrapyrrole ring [14]. Pathogenic bacteria use several strategies to acquire iron, including import of Fe^2+^ by ATP- or GTP-dependent inner membrane transporters and TonB-ExbB-ExbD-dependent transport of ferric siderophores, transferrins, and heme or heme-bound proteins through specific outer membrane receptors [15]. The impact of Mn^2+^ on biofilm formation by *Bacillus subtilis* is well established [7,16]. Recently, it was reported that a combination of glycerol and Mn^2+^ can also trigger *B*. *cereus* biofilm formation [7,8]. Comparative genomic studies of iron transport mechanisms have revealed that iron intake significantly affects *B*. *cereus* biofilm formation [5]. However, the effects of Mn^2+^ and heme on biofilm formation-related phenotypes, including cell density, sporulation, motility and gene expression, are not well understood.

In *B*. *subtilis*, biofilm formation is dependent on the level of phosphorylation of Spo0A (Spo0A~P), which is mainly controlled by a network of histidine kinases [17,18]. Low levels of Spo0A~P induce a network for expression of EPS, leading to biofilm formation [17]. Exposure of *B*. *subtilis* to a combination of Mn^2+^ and glycerol in LB decreases the activation of histidine kinase HinD, which reduces Spo0A~P levels and subsequently activates the gene expression cascade responsible for EPS production [8]. For instance, Spo0A~P triggers the upregulation of SinI, an anti-repressor, which in turn activates the central biofilm master repressor SinR [19]. Homologs of *spo0A*, *sinI* and *sinR* of *B*. *subtilis* are also present in *B*. *cereus* and are known to play an important role in biofilm formation [20]. In *B*. *subtilis*, SinR directly regulates the activities of *tapA-sipW-tasA* and *eps* operons which are responsible for biofilm matrix formation [21]. The *tapA-sipW-tasA* operon of *B*. *subtilis* is closely related to the *sipW-tasA* loci that is required for biofilm formation in *B*. *cereus* [22]. However, in a recent study, the homologous of *eps* operon of *B*. *subtilis* has shown not to be important for biofilm matrix formation in *B*. *cereus* [6]. In addition, the regulator gene *abrB* along with *sp0A* suppresses the expression of a cascade of genes involved in motility and sporulation of *B*. *subtilis* [23]. However, the role of *spo0A* and *abrB* in *B*. *cereus* biofilm formation-related characteristics is presently unclear.

Because *B*. *cereus* forms biofilms in particular under nutrient-rich conditions such as those that exist in the food processing industry, it is important to identify the molecules that might induce biofilm formation. Therefore, we performed this study to understand the impact of Mn^2+^ and heme on biofilm formation and other biofilm-related phenotypes in several *B*. *cereus* food isolates.

## Materials and methods

### Strains and culture conditions

A total of six *B*. *cereus* strains isolated from Korean soybean paste, including two reference strains, were used in this study (Table 1). Stock cultures were stored at -80°C in brain heart infusion (BHI) (Becton Dickinson, Sparks, USA) containing 15% glycerol (Daejung, Busan, Korea). The stock cultures were routinely grown on BHI agar plates and incubated at 30°C for 24 h to prepare working cultures. Overnight (18 h) broth cultures were inoculated from single colonies at 30°C. The OD at 600 nm of the culture was measured to maintain an approximate cell concentration of 7 logCFU/ml of each strain in buffered peptone water (BPW). These cultures were used in all further experiments.

**Table 1:** List of *B*. *cereus* strains used in this study.

*B*. *cereus* strain*	Type of strain	Reference
**ATCC 14579**	Reference	[37]
**ATCC 10987**	Reference	[37]
**GIHE 72–2**	Soybean paste	This study
**GIHE 72–4**	Soybean paste	This study
**GIHE 72–5**	Soybean paste	This study
**GIHE 72–6**	Soybean paste	This study
**GIHE 72–7**	Soybean paste	This study
**GIHE 72–8**	Soybean paste	This study

*ATCC, American Type Culture Collection; GIHE, Gangwon Institute of Health and Environment.

### Biofilm formation

Static biofilms were formed on stainless steel (SS) (AISI type 304L) (18 x 18 mm) coupons placed vertically in the wells of a 12-well microtiter plate (SPL LifeSciences, Gyeonggi, South Korea) as described previously by Hayrapetyan et al. [24]. The SS coupons were pretreated to remove dirt and other organic compounds using the method described by Castelijn et al. [25]. Biofilms were grown in BHI because this medium promotes higher biofilm formation than other media for several *B*. *cereus* food isolates [24]. Manganese sulfate (Mn^2+)^) (Sigma, St. Louis, USA) and heme (Sigma, St. Louis, USA) at final concentrations of 0.005% and 10 μg/ml, respectively were added to BHI broth where indicated [26]. In addition, BHI was supplemented with either glucose (Merck, Darmstadt, Germany) or glycerol to a final concentration of 2% [6]. Each well of the microtiter plate containing an SS coupon was filled with 3 ml of BHI supplemented with Mn^2+^, heme, glucose and glycerol alone or in combination. The BHI broth was then inoculated with 1.0% volume of an overnight culture. The plates were incubated at 30°C for 48 h under static conditions.

### Biofilm quantification

The crystal violet assay described previously by Castelijn et al. [25] was used to measure biofilm formation. Briefly, after incubation, the SS coupons were carefully washed three times by dipping into phosphate-buffered saline (PBS) (Life Technologies, Grand Island, USA) using sterile forceps [24]. The attached biofilms were stained with 0.1% crystal violet (Difco, Detroit, USA) for 30 minutes. Crystal violet that did not bind to biofilms was discarded. The coupons were washed again three times with PBS and incubated in 70% ethanol for 30 min to release the biofilms bound by the crystal violet. Solubilized crystal violet was quantified by measuring the absorbance at a wavelength of 595 nm (Molecular Devices, Berkshire, UK). crystal violet assays were performed in three independent experiments.

The number of cells in biofilms on SS coupons incubated with isolate GIHE 72–5 was determined using a previously published protocol [25]. In brief, the coupons were dipped into sterile PBS three times. The coupons were then transferred to a tube containing 10 ml PBS and 0.5 g sterile glass beads (<106 μm, Sigma, St. Louis, USA). The coupons with attached cells were vortexed at maximum speed for 1 min, the resulting suspension was transferred to a new 96-well plate, and appropriate serial dilutions were made in PBS [24]. One hundred microliters of the serially diluted samples were spread on BHI agar plates and incubated at 30°C for 24 h. Subsequently, the number of colonies was counted and expressed as logCFU/cm^2^. Three independent sets of cell enumeration experiments were performed.

The number of spores in the biofilms was measured using a modification of published protocols [24]. GIHE 72–5 biofilms were grown on plastic SS coupons in BHI supplemented with Mn^2+^, heme and glycerol alone or in combination in 12-well plates at 30°C for 48 h. After incubation, the medium was removed, the coupons were washed with PBS, and the attached cells were separated as described in previous section. The suspension was heated at 80°C for 10 min to inactivate vegetative cells. One hundred microliters of the spore suspension was spread on BHI agar plates followed by incubation at 30°C for 24 h. Subsequently, the number of colonies was enumerated in logCFU/cm^2^. At least three independent experiments were performed to determine the number of spores in the biofilm matrix.

### Scanning electron microscopy

*B*. *cereus* GIHE 72–5 biofilms formed on SS coupons were prepared for SEM imaging as described previously [25]. Briefly, biofilms on SS coupons were fixed in 3% glycerol for 1 h. The coupons were rinsed three times with PBS, treated with osmium tetroxide (TCI, Tokyo, Japan) for 1 h, washed twice with distilled water and dehydrated using an acetone series (10%, 30%, 50%, 70%, 90% and 100%) with a 15-min incubation at each acetone concentration. The samples were then critical-point dried using carbon dioxide. For image analysis, the specimens were sputter-coated with iridium, and images were acquired using an FESEM electron microscope (Model 5430, Hitachi, Tokyo, Japan). SEM image analysis was performed in two independent experiments.

### Confocal laser scanning microscopy

The live/dead status of GIHE 72–5 cells in the biofilms was investigated using confocal laser scanning microscopy (CLSM). Biofilms were formed on plastic coupons (18 x 18 mm) (Rinzl, Electron Microscopy Sciences, PA, USA) placed vertically in the wells of a 12-well microtiter plate containing BHI supplemented with Mn^2+^, heme and glycerol alone or in combination as described in the previous section. After incubation, the coupons were stained with 4 ml of LIVE/DEAD Viability kit reagent (Invitrogen, MA, USA) in saline (0.9% NaCl) containing a mixture of SYTO-9 and propidium iodide (1.5 μl/ml of each) and incubated for 15 minutes in the dark [27]. CLSM was then performed using a Zeiss LSM710 microscope (Carl Zeiss Microscopy GmbH, Jena, Germany) equipped with excitation lasers at 488 nm and 514 nm and an EC Plan-Neofluar 40x /1.30 oil lens. Five microscopic fields from each coupon were randomly recorded for image acquisition. Two CLSM image analysis experiments were performed.

### Benzalkonium chloride resistance

Biofilms of selected *B*. *cereus* food isolate GIHE 72–5 formed on SS were treated with disinfectant benzalkonium chloride (BAC) at 200 ppm according to published method of Poimenidou et al. [28]. Briefly, biofilms on SS slides in BHI were incubated at 30°C for 48 h. After incubation, SS was washed twice by dipping twice into PBS to remove loosely attached cells. Subsequently, SS was transferred to a 50-ml tube filled with 10 ml of BAC. Following 5 min exposure to BAC, biofilm cells were separated by vortexing vigorously with beads for 1 min with maximum speed on a vortex. Then 1 ml of biofilm suspension was transferred to a new tube filled with 9 ml of D/E Neutralizing Broth (Becton, Dickinson and Company, NJ, USA). Neutralized samples were then used for cell enumeration as described previously.

### Swarming motility test

The swarming mobility of GIHE 72–5 planktonic cells was measured according to Yan el al. [8]. Briefly, an overnight culture was grown in BHI supplemented with heme, Mn^2+^, and glycerol alone or in combination at 30°C for 18 h. One milliliter of this culture was washed twice with PBS and resuspended in 100 μl of PBS. Five microliters of the resuspension was spotted on BHI soft agar (0.5% agar), and the plate was dried in a laminar hood for 10 min. The swarming plates were incubated at 37°C for 12 h. Subsequently, the plates were dried for one hour in a laminar flow hood and incubated for 12 h at room temperature prior to measurement of the swarming zone. Swarming motility was tested in at least three independent experiments.

### RNA preparation and quantitative RT-PCR

Total RNA was extracted from *B*. *cereus* GIHE 72–5 biofilms formed on SS in BHI supplemented with heme, Mn^2+^ and glycerol at 30°C for 48 h using an RNeasy Mini kit (Qiagen, Hilden, Germany) according to the manufacturer’s instructions. Total RNA was also extracted from planktonic overnight cultures grown in BHI supplemented with heme, Mn^2+^ and glycerol alone or in combination at 30°C for 18 h. RNA quantity was measured using a NanoDrop spectrophotometer. cDNA samples were prepared using a SuperScriptH III Reverse transcriptase kit (Invitrogen, MA, USA) according to the manufacturer’s instructions. Real-time PCR measurements were performed using an ABI System (Applied Biosystems Inc, CA, USA) as described previously [29]. The primers’ sequences used in this study were selected from previous study and are shown in Table 2. The 16S rRNA sequence was used as an internal standard. RT-PCR was performed in three independent experiments with three to five replicates in each experiment.

**Table 2:** List of primers used in real-time PCR.

**Gene**	**Primer**	**Sequence (5’ to 3’)**	**Source**
**16SrRNA**	16SrRNA_F	GGAGGAAGGTGGGGATGACG	[38]
	16SrRNA_R	ATGGTGTGACGGGCGGTGTG	
***abrB***	abrB_F	TCGTGTAGTAATTCCGATTGA	[38]
	abrB_R	TGAAGCTCGTTTAAGATTTGC	
***spoOA***	spoOA_F	GAAGATCTACTCCACAAAAAGACAACGGTG	[6]
	spoOA_R	CGACGCGTGCCGTTCCTTCATCATTTAATA	
***sinI***	sinI_F	CATGCCATGGAGGAACATTTGCATTCTTTAGC	[6]
	sinI_R	CGACGCGTCTAATTTTTCTTTCGTGTCTGC	
***sinR***	sinR_F	GAAGTAGAGTATCAACTGGA	[8]
	sinR_R	GCTGGTGTTGCTAAATCTTAC	
***tasA***	tasA_F	AGCAGCTTTAGTTGGTGGAG	[22]
	tasA_R	R GTAACTTATCGCCTTGGAATTG	
***sipW***	sipW_F	AGATAATTAGCAACGCGATCTC	[22]
	sipW_R	AGAAATAGCGGAATAACCAAGC	

### Statistical analysis

The data shown are average values obtained in at least three independent experiments with standard deviations. For assessment of significant differences, one-way analysis of variance (ANOVA) with Tukey's post hoc test was performed using SPSS (IBM SPSS Statistics, version 22, USA). Statistical significance was considered when the *p* value was less than 0.05.

## Results

### Impact of Mn^2+^ and heme on biomass formation by *B*. *cereus* food isolates

**Fig 1:**
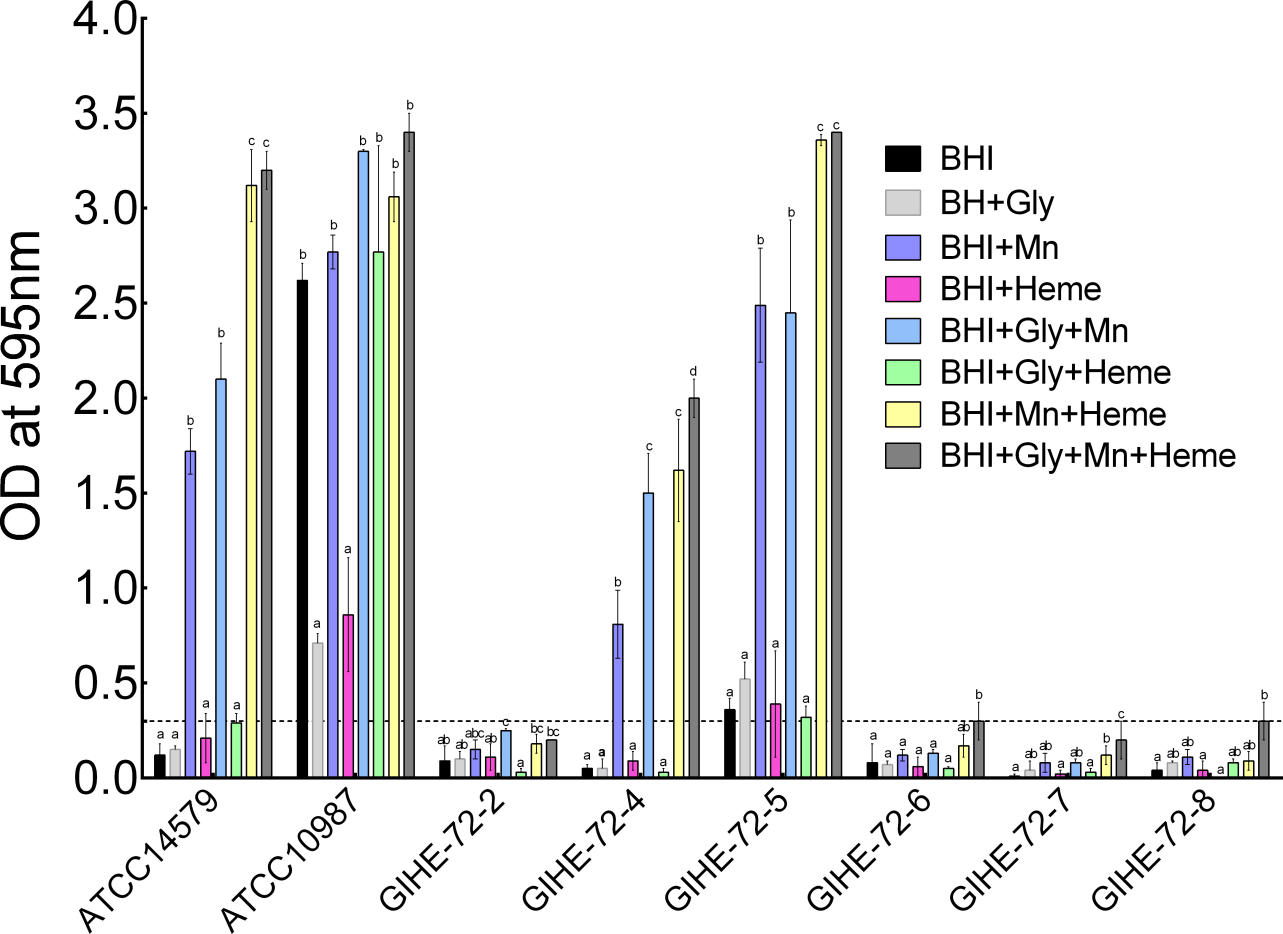
Impact of Mn^2+^ and heme on biofilm formation by *B*. *cereus* food isolates. Biofilms were grown on SS coupons with or without Mn^2+^ and/or heme in BHI supplemented with glycerol at 30°C for 48 h. Established biofilms were quantified using the crystal violet assay. The threshold of biofilm formation (solid line) is equal to the background absorbance value plus three times the standard deviation (OD = 0.3). Each data point represents the average value obtained in three biological experiments for each strain. The error bars indicate the standard deviation. To compare effects on biofilm formation in each strain, one-way ANOVA and Tukey's post hoc test (p < 0.05) were performed. Groups marked with different letters in each strain display significant differences.

The impact of Mn^2+^ and heme on the biofilm formation capacity of six *B*. *cereus* food isolates and two reference strains (ATCC 14579 and ATCC 10987) was investigated. Biofilms were grown on SS coupons with or without Mn^2+^ and/or heme in BHI supplemented with glucose or glycerol. The results are shown in Fig 1. In the crystal violet assay, addition of Mn^2+^ to BHI medium (BHI+Mn) dramatically promoted biofilm formation by three of the strains under almost all conditions tested; the responding strains included one of the reference strains (ATCC 14579) and two of the food isolates (GIHE 72–4 and GIHE 72–5). After the addition of Mn^2+^ and heme to BHI (BHI+Mn+Heme), more biofilms were grown by these strains than after the addition of Mn^2+^ alone (BHI+Mn). In Mn^2+^- and heme-containing BHI supplemented with glycerol (BHI+Gly+Mn+Heme), a large amount of biofilm was produced only by the GIHE 72–4 isolate. Although higher crystal violet values were obtained for the GIHE 72–6, GIHE 72–7 and GIHE 72–8 isolates grown in BHI supplemented with Mn^2+^, heme and glycerol (BHI+Gly+Mn+Heme), the values were lower than the threshold values for biofilm formation (OD ≤ 0.3 at 595 nm). Growth of *B*. *cereus* in Mn^2+^- and heme-containing BHI supplemented with glycerol promotes biofilm formation on SS coupons. However, under conditions of supplementation of BHI with excess glucose in the presence of Mn^2+^ and/or heme (BHI+Glc+Mn+Heme), substantial biofilm formation on SS coupons did not occur (S1 Fig). Moreover, addition of heme alone to BHI or supplementation of BHI with heme and glycerol (BHI+Gly+Heme) had no impact on biofilm formation in any of the *B*. *cereus* strains tested.

Because the results of the crystal violet assays showed that growth of the *B*. *cereus* food isolate GIHE 72–5 in BHI medium containing added Mn^2+^ and heme dramatically induced robust biofilm formation (Fig 1), this strain was selected for further experiments in which the impact of Mn^2+^ and heme on *B*. *cereus* biofilm formation was studied.

### Impact of Mn^2+^ and heme on the number of cells and spores in biofilms of *B*. *cereus*

**Fig 2:**
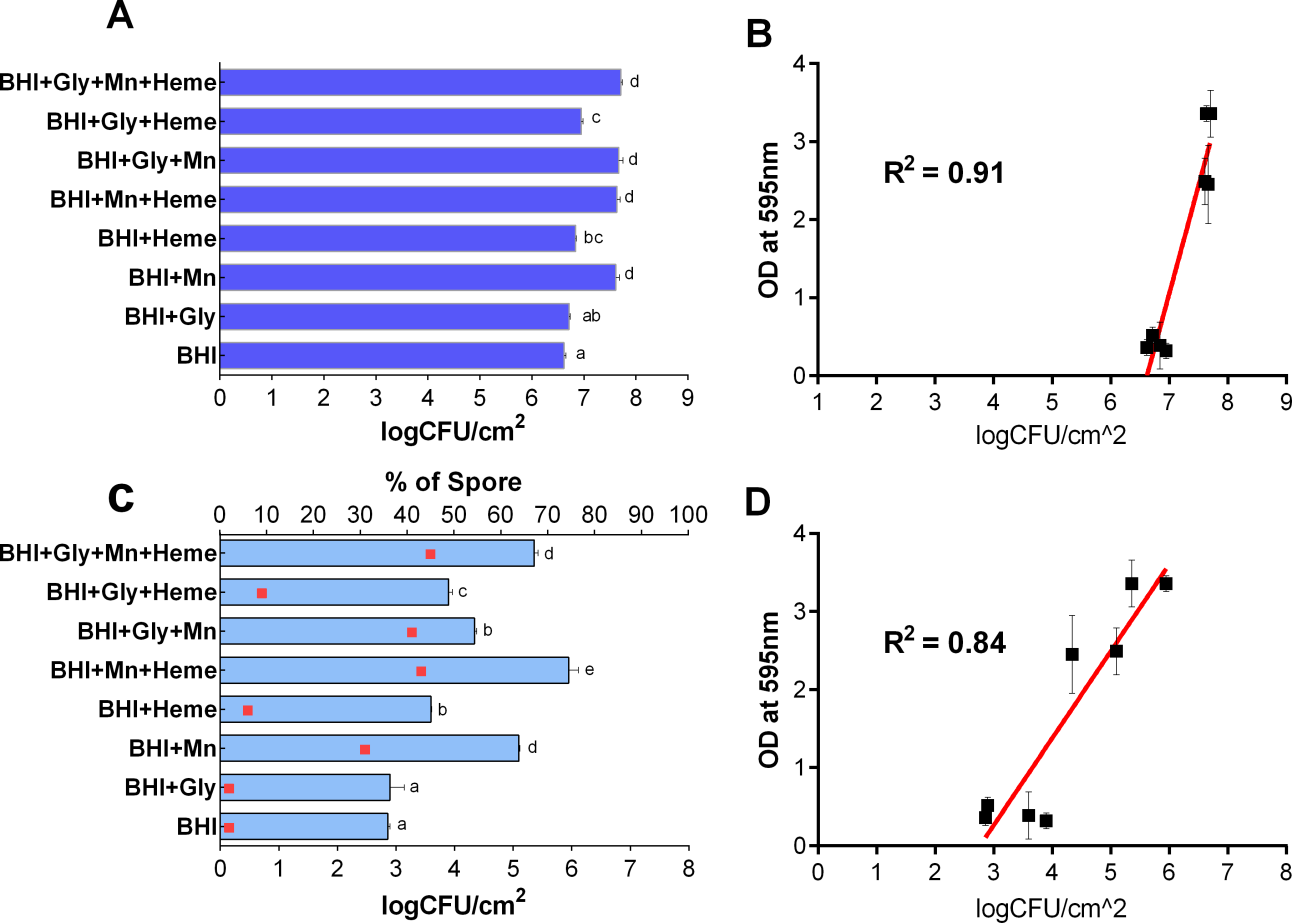
Number of cells and spores in *B*. *cereus* biofilms. *B*. *cereus* GIHE 72–5 was grown in BHI supplemented with glycerol with or without Mn^2+^ and heme at 30°C for 48 h. Number of cells in logCFU/cm^2^ (A); scatter plot showing the relationship between the number of cells and the results of crystal violet assays (B); number of spores in logCFU/cm^2^ (C); scatter plot showing the relationship between spore formation and the results of crystal violet assays (D). While, squares (C) show the percentage of spore formation compared to the number of cells in biofilms. Each data point represents the average value obtained in three biological experiments, and the standard deviation. To compare the number of cells and spores obtained among the conditions used, one-way ANOVA and Tukey's post hoc test (p < 0.05) were performed. Groups marked with different letters in each growth condition display significant differences.

The impact of Mn^2+^ and heme on the number of cells within the biofilm matrix was determined for the selected *B*. *cereus* GIHE 72–5 isolate using cell enumeration. For these experiments, biofilms were grown with or without Mn^2+^ and/or heme in BHI supplemented with glycerol on SS coupons at 30°C for 48 h. The results are shown in Fig 2. The largest number of cells (p < 0.05) was found in biofilms grown in Mn^2+^- and heme-containing BHI supplemented with glycerol (BHI+Gly+Mn+Heme), as shown in Fig 2A. Significantly greater (p < 0.05) numbers of cells were present in biofilms grown in BHI supplemented with Mn^2+^ (BHI+Mn) than in biofilms grown in BHI alone. However, there was no significant (p > 0.05) difference in the number of cells in biofilms grown in BHI containing Mn^2+^ and heme (BHI+Mn+Heme) and those grown in BHI supplemented only with Mn^2+^ (BHI+Mn). The correlation between total biomass formation measured in the crystal violet assay and the number of cells in biofilms obtained with or without Mn^2+^ and/or heme supplementation of BHI was calculated. In scatter plots (Fig 2B), the number of cells and the OD values obtained from the crystal violet assay showed a good correlation (R^2^ = 0.91). Notably, the impact of Mn^2+^ and heme on the planktonically grown cells was also determined for the selected *B*. *cereus* GIHE 72–5 isolate using cell enumeration. However, no correlation was found between planktonically grown cells and biofilm formation by addition of Mn(II) and Heme (S2 Fig). Sporulation of GIHE 72–5 in biofilms grown on SS coupons at 30°C for 48 h was determined in the presence and absence of Mn^2+^ and/or heme supplementation of BHI. A higher degree of sporulation was seen in biofilms grown in BHI supplemented with either Mn^2+^ or heme (BHI+Mn^2+^ or BHI+Heme) than in BHI alone (Fig 2C). However, under almost all conditions, higher sporulation in biofilms was found after the addition of Mn^2+^ than after the addition of heme to BHI. The maximum number of spores occurred in biofilms grown in BHI supplemented with both Mn^2+^ and heme (BHI+Mn+Heme). Notably, in biofilms grown in Mn^2+^- and heme-supplemented BHI (BHI+Mn+Heme), the spore-forming efficacy (the number of spores formed compared to the number of cells) was as high as 43%, whereas in BHI alone it was only 2%. The linear coefficient value (R^2^) for the correlation between the number of spores formed and OD values obtained from the crystal violet assay was 0.84, indicating good correlation between spore formation and biofilm formation (Fig 2D).

### SEM analysis of *B*. *cereus* biofilms grown in BHI with added Mn^2+^ and heme

**Fig 3:**
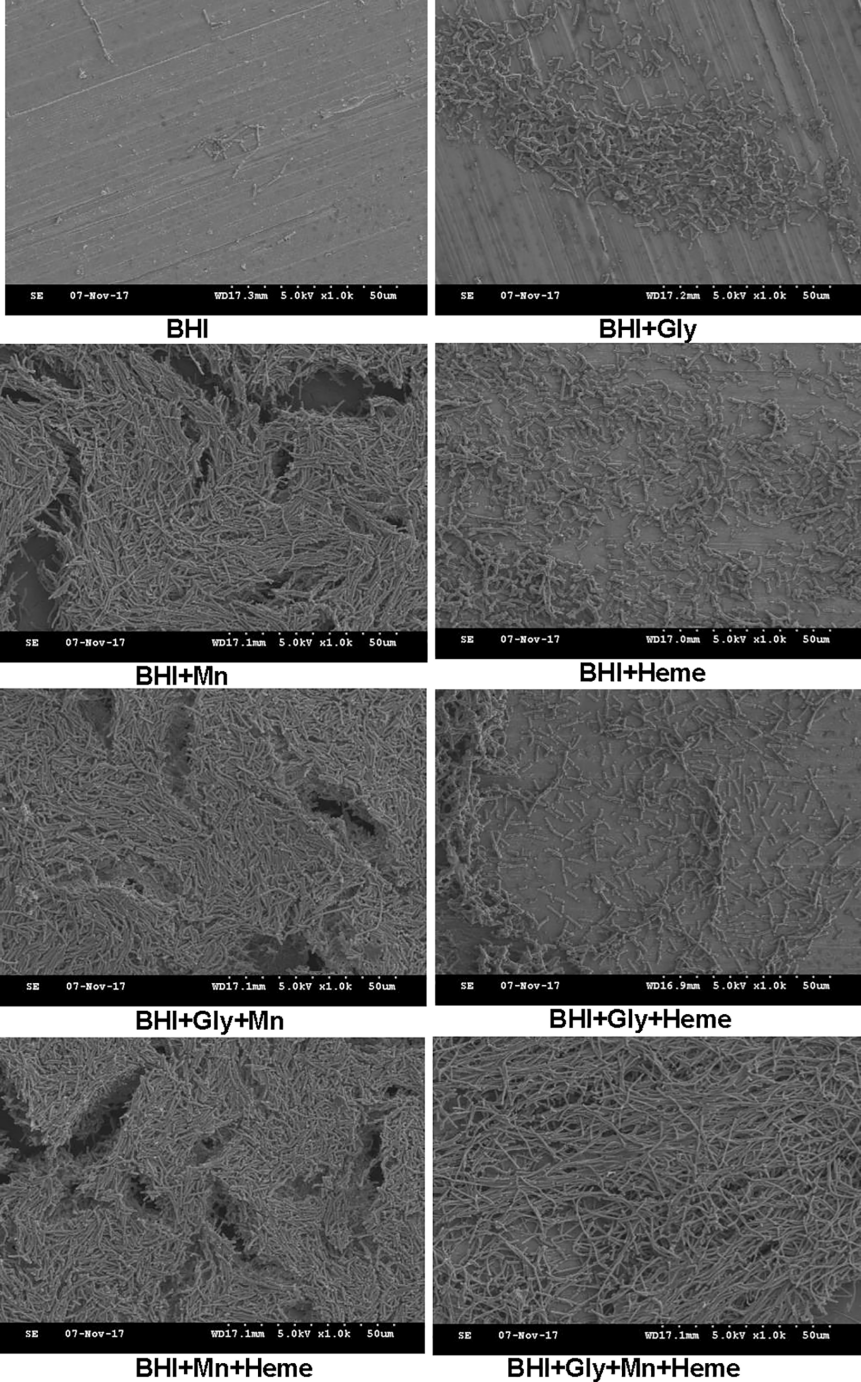
Representative scanning electron microscope (SEM) images of biofilms formed by *B*. *cereus* GIHE 72–5.

The biofilm morphology, cell heterogeneity and structure of the selected *B*. *cereus* food isolate GIHE 72–5 were visualized by SEM analysis. Biofilms grown on SS coupons in Mn^2+^-supplemented BHI displayed a complex structure with greater numbers of cell clusters compared to other conditions (Fig 3). Interestingly, in biofilms produced in BHI containing added Mn^2+^, heme and glycerol (BHI+Gly+Mn+Heme), a complex structure with relatively longer cell clusters was observed. SEM analysis revealed consistent results; in particular, biofilms grown in Mn^2+^-supplemented BHI, which yielded higher values in the crystal violet and cell enumeration assays, also contained higher numbers of cells within the biofilms.

### Viability of cells within *B*. *cereus* biofilms grown in BHI with added Mn^2+^ and heme

**Fig 4:**
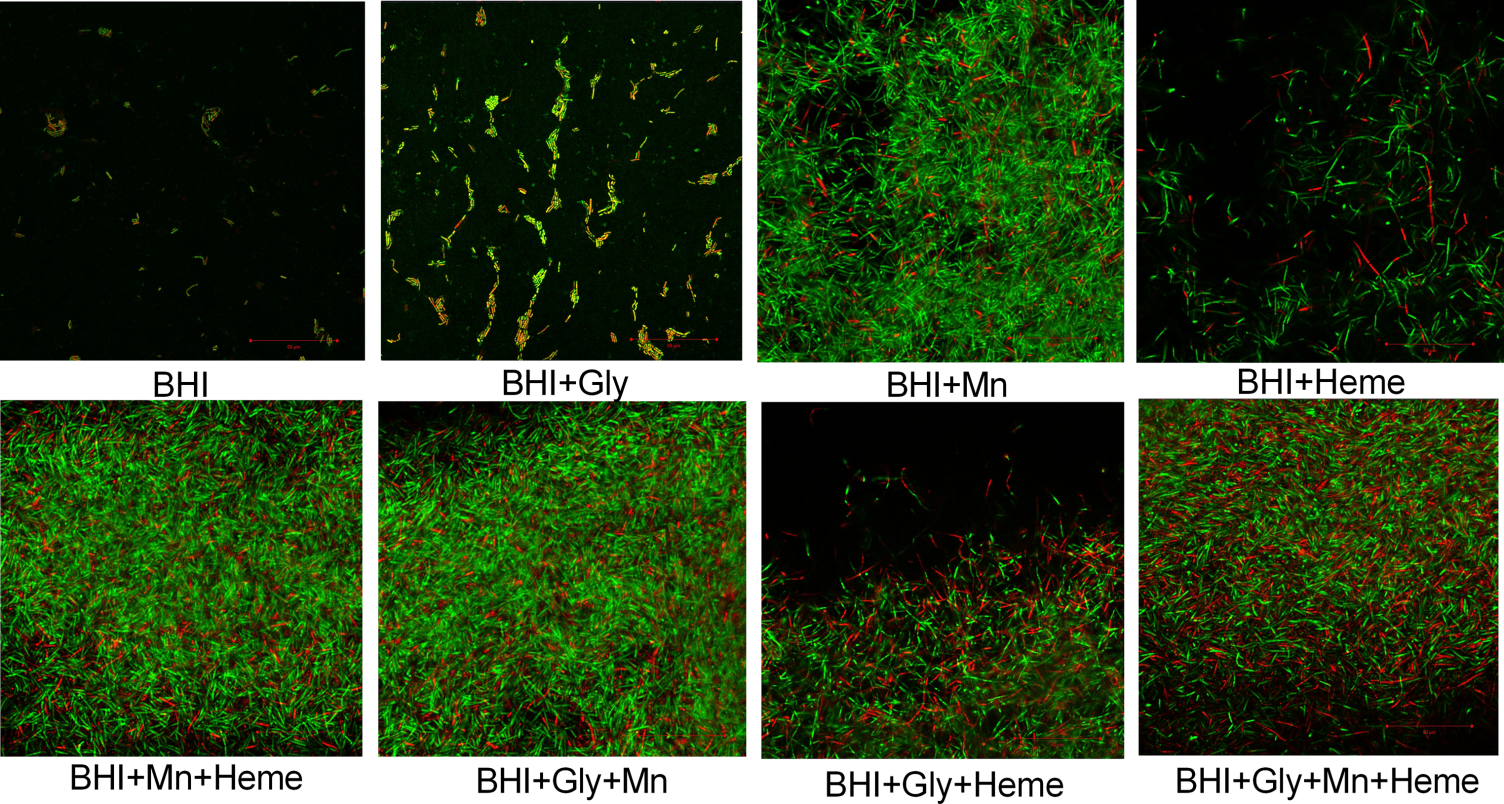
Representative confocal laser scanning microscopy (CLSM) images of biofilm formation by *B*. *cereus* GIHE 72–5. *B*. *cereus* GIHE 72–5 biofilms were grown on plastic coupons with or without Mn^2+^ andr heme in BHI supplemented with glycerol at 30°C for 48 h. Subsequently, the biofilms were stained using a LIVE/DEAD BacLight bacterial viability staining kit. The scale bar represents 50 μm.

The viability of *B*. *cereus* GIHE 72–5 cells within biofilms grown on plastic coupons in BHI with or without Mn^2+^ and/or heme supplementation was assessed using LIVE/DEAD staining. In biofilms grown in Mn^2+^-supplemented BHI with or without the addition of glycerol, most cells were stained green, indicating that the cells were viable (Fig 4). In contrast, a high number of the cells in biofilms grown in heme-supplemented BHI were stained red, indicating that the membranes of these cells were compromised during biofilm formation. Moreover, in biofilms grown on plastic coupons in BHI with added Mn^2+^, a greater number of cell clusters was found compared to other conditions (Fig 4). CLSM analysis of biofilms grown on plastic coupons showed consistent results; in particular, biofilms grown in Mn^2+^-supplemented BHI showed higher values in the crystal violet assay and higher numbers of cells in cell enumeration experiments (S3 Fig).

### Swarming ability of *B*. *cereus* planktonic cells grown in BHI containing Mn^2+^ and heme

**Fig 5:**
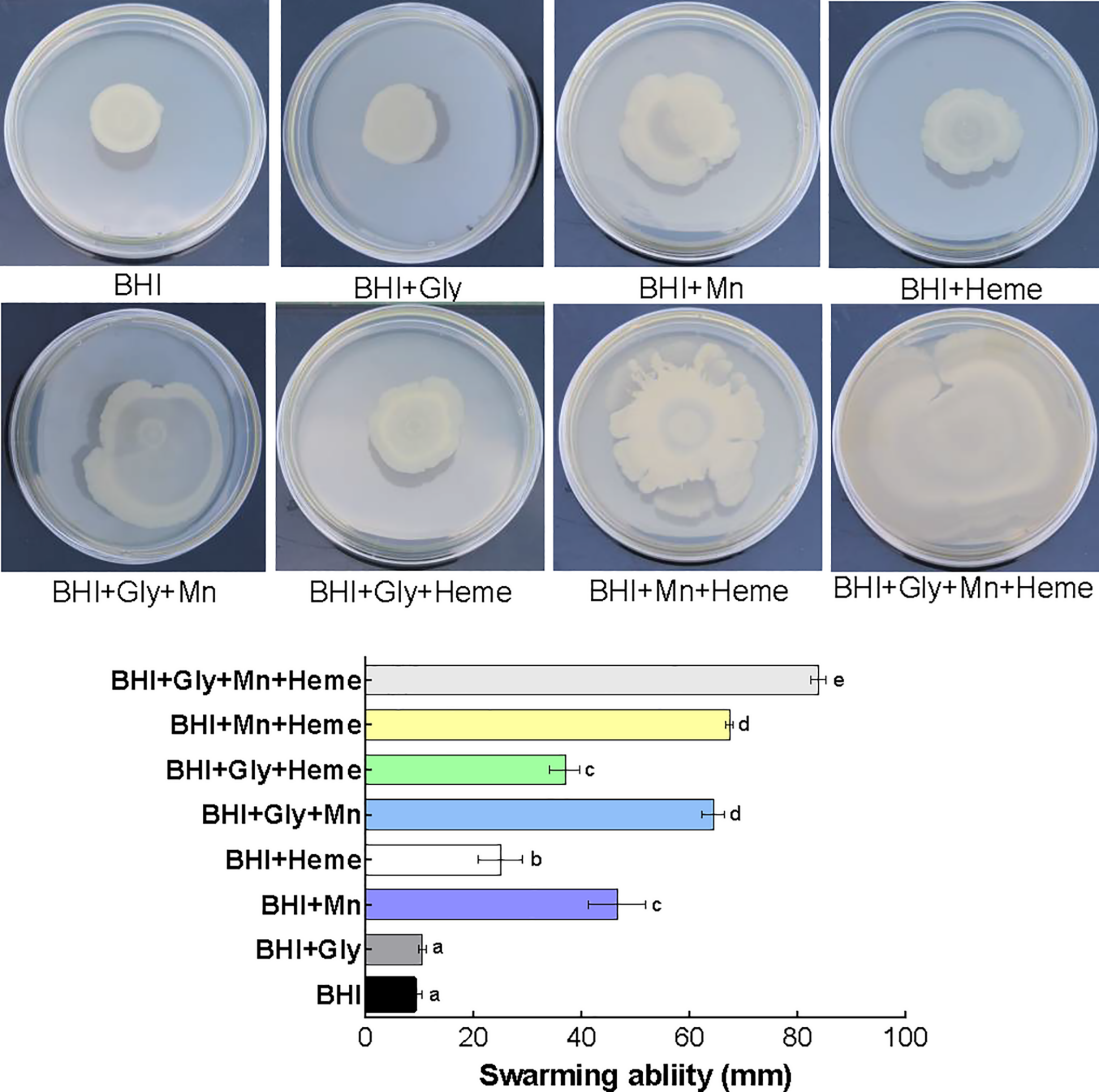
Swarming motility of *B*. *cereus* food isolate GIHE 72–5 planktonic cells. Planktonic cells were grown in BHI with or without Mn^2+^ and/or heme in BHI supplemented with glycerol at 30°C overnight (18) h. The overnight cultures were washed in PBS, and their swarming mobility on BHI soft agar (0.5%) plates was examined. The data represent the average swarming mobility obtained in three biological experiments; the standard deviation is shown. To compare the swarming mobility under different conditions, one-way ANOVA and Tukey's post hoc test (p < 0.05) were performed. Groups marked with different letters display significant differences.

*B*. *cereus* GIHE 72–5 planktonic cells were tested for their swarming ability after growth in Mn^2+^- and/or heme-supplemented BHI to determine whether this property might be related to growth conditions and/or to subsequent biofilm formation. Swarming zones were measured on BHI soft agar (0.5%). Planktonic cells grown in BHI with added Mn^2+^ showed higher swarming ability under almost all conditions (Fig 5). The largest swarming zone was observed after growth of the cells in BHI supplemented with Mn^2+^, heme and additional glycerol (BHI+Gly+Mn+Heme). Importantly, the results of crystal violet assays as well as those of cell enumeration assays indicated that high amounts of biofilms were formed on SS coupons under these growth conditions.

### Impact of Mn^*2+*^ and heme in BHI on the resistance of *B*. *cereus* biofilm cells to benzalkonium chloride (BAC)

**Fig 6:**
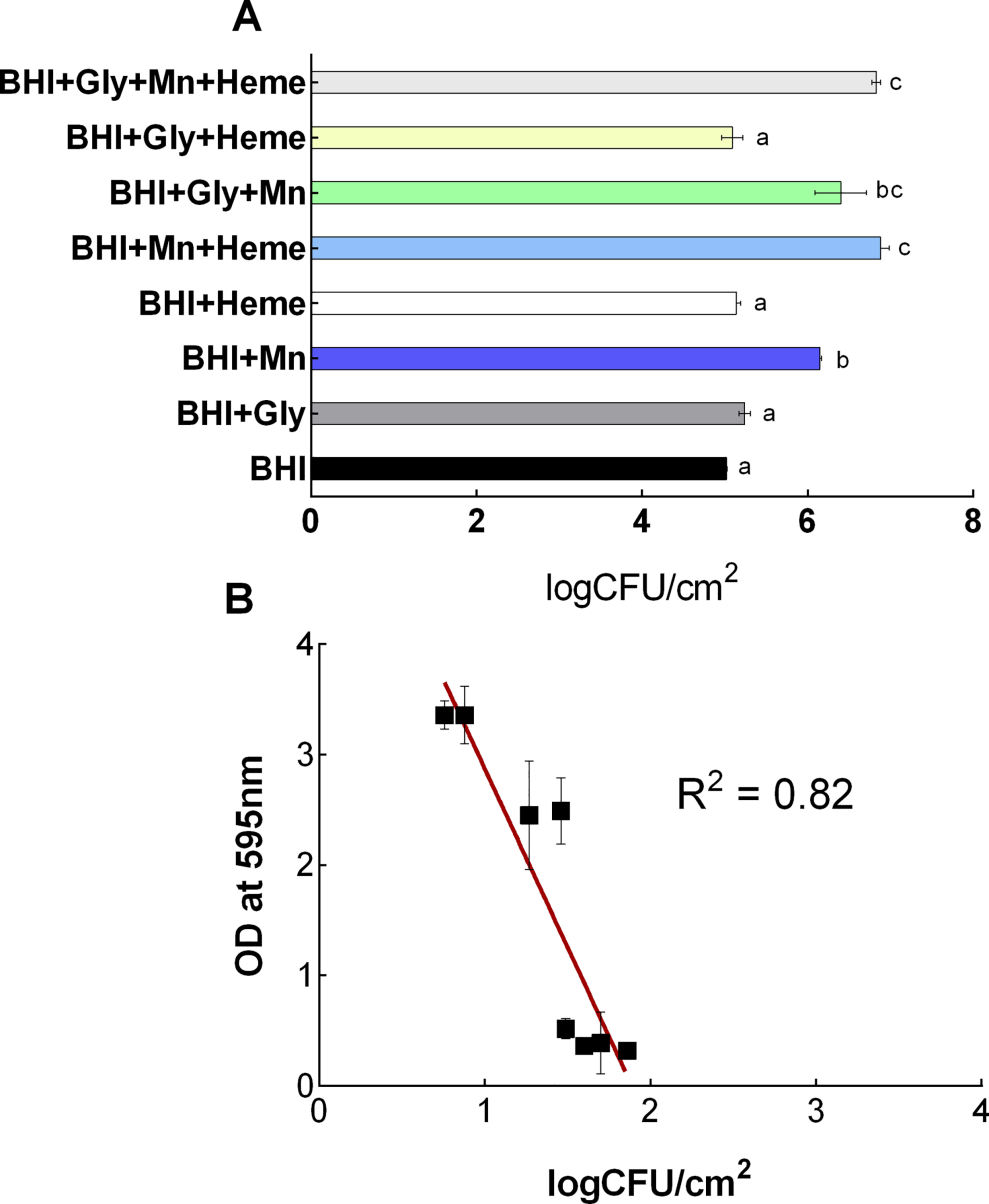
Impact of Mn^2+^ and heme on the resistance of *B*. *cereus* biofilm cells to benzalkonium chloride. Biofilms of the *B*. *cereus* food isolate GIHE 72–5 were grown on SS coupons with or without Mn^2+^ and/or heme in BHI supplemented with glycerol at 30°C for 48 h. After maturation, biofilms were washed with PBS and subsequently treated with BAC (200 μg/ml) for 5 min, and the number of surviving cells was determined (A). The average number of surviving cells in logCFU/cm^2^ (A) is shown as a scatter plot of the Log reduction versus the crystal violet assay results (OD values) (B). Each data point represents the average value obtained in three biological experiments; the standard deviation for each condition is shown. To compare the number of surviving cells obtained under different conditions, one-way ANOVA and Tukey's post hoc test (p < 0.05) were performed. Groups marked with different letters display significant differences.

The resistance of biofilms grown in Mn^2+^- and/or heme-supplemented BHI to BAC of the selected *B*. *cereus* GIHE 72–5 isolate was determined. The results showed that the biofilms grown on SS coupons in Mn^2+^-supplemented BHI, in almost all cases, displayed higher resistance to exposure to BAC (200 μg/ml) for 5 min (Fig 6). For example, in biofilms grown in BHI supplemented with Mn^2+^, heme, and glycerol, the number of cells within the biofilm matrix was reduced by only 0.88 logCFU/cm^2^ after BAC treatment, whereas the number of cells was reduced by 1.6 logCFU/cm^2^ when biofilms grown in BHI alone were exposed to the same treatment.

### Impact of Mn^2+^ and heme on the expression of *spoOA* and *abrB* during the planktonic and biofilm growth phases of *B*. *cereus*

**Fig 7:**
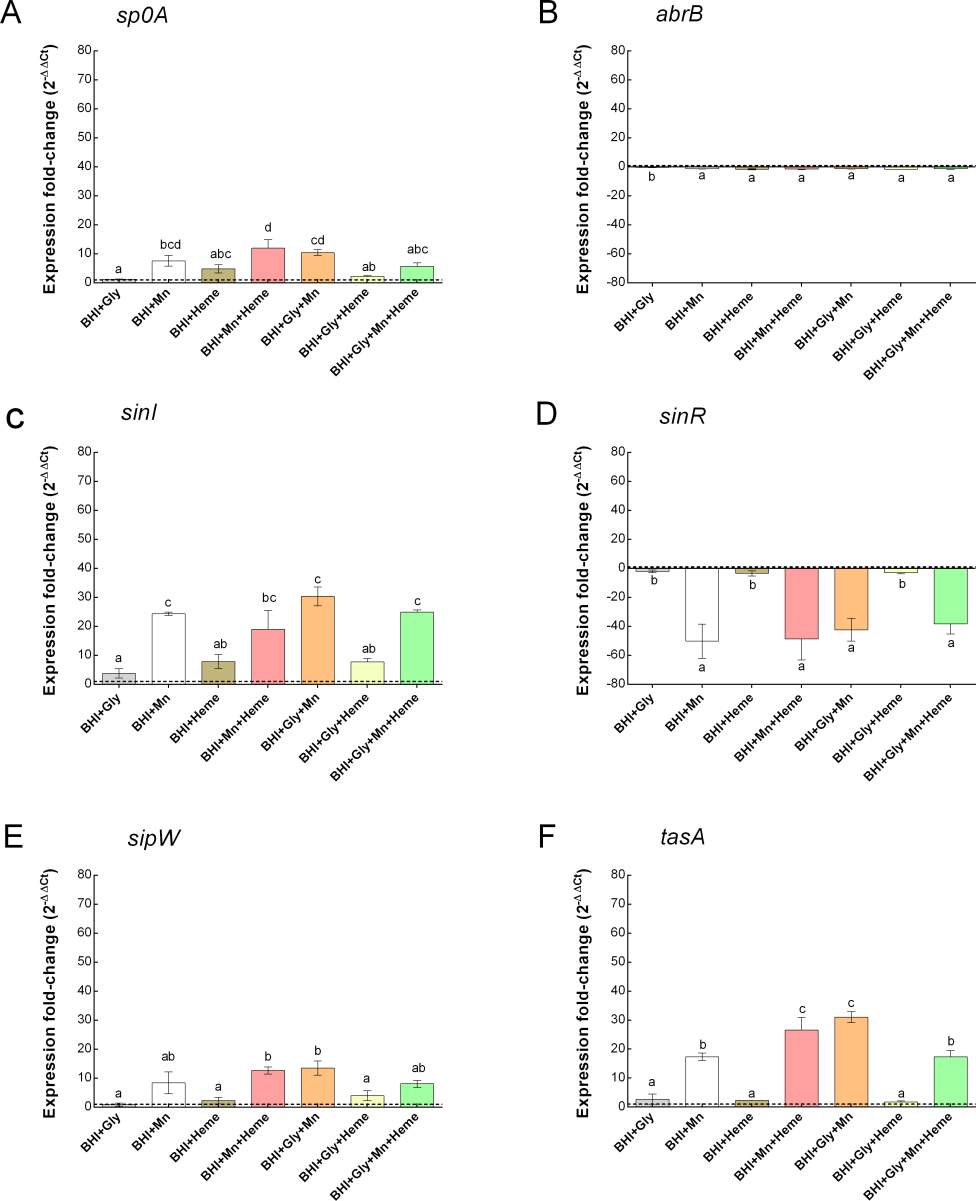
Impact of Mn^2+^ and heme on the expression of a number of selected genes in *B*. *cereus* in the biofilm growth phases. The graphs show the expression of *spoOA* (A), *abrB* (B), *sinI* (C), *sinR* (D), *sipW* (E) and *tasA* (F) in the biofilm phase of GIHE 72–5. Biofilms were grown on SS coupons in Mn^2*+*^ and/or heme in BHI supplemented with glycerol at 30°C for 48 h. The fold change in expression relative to the expression in biofilm cells grown in BHI is shown. Each data point represents the average value obtained in at least two biological experiments; the error bars indicate standard deviation. To compare fold changes in expression under different conditions, one-way ANOVA and Tukey's post hoc test were performed. Groups marked with different letters in each growth condition display significant differences (p < 0.05).

The effects of Mn^2+^ and heme supplementation of BHI on the expression of selected genes, *spoOA*, *abrB*, *sinI*, *sinR*, *sipW* and *tasA* during planktonic and biofilm growth conditions of GIHE 72–5 were tested. In general, *spoOA*, *sinI*, *sipW and tasA* were upregulated in BHI supplemented with Mn^2+^ under almost all conditions tested in the biofilm growth phases (Fig 7) compared to growth in BHI alone. Whereas, *abrB* and *sinR* were downregulated under this conditions (Fig 7B and 7D). Meanwhile, all of the tested genes were upregulated except *sinR*, which was downregulated, during planktonic growth conditions (S3 Fig). Maximal expression of *spo0A*, *sinI* and *tasA* were observed in biofilm cells grown in BHI supplemented with glycerol, Mn^2+^ and/or heme (BHI+Gly+Mn) or (BHI+Gly+Mn+Heme). In contract, *sinR* was minimally downregulated under these conditions. Importantly, there was no significant (p > 0.05) difference among the tested biofilm growth conditions for the downregulation of *abrB* (Fig 7B). In the planktonic growth phase, although, *sp0A*, *sinI*, *sipW* and *tasA* were upregulated but there were no significant (p > 0.05) differences during addition of Gly, Mn, and Heme in BHI (S4 Fig). Like *spo0A*, *sinI*, *sipW* and *tasA*, *abrB* was also upregulated but significantly (p < 0.05) higher after the addition of Mn^2+^ to BHI under almost all conditions tested in the planktonic growth phase (Fig 7B).

## Discussion

In this study, we demonstrated that Mn^2+^ supplementation dramatically induces *B*. *cereus* biofilm formation during growth in BHI medium. The combination of glycerol and Mn^2+^ has been reported previously to induce *B*. *cereus* biofilm formation in LB growth medium [7,8]. Genome-wide transcriptome analysis shows that addition of glycerol and Mn^2+^ to LB results in a metabolic shift that leads to increased fermentation and higher production of small fermentation products (i.e., acetone, lactate, and ethanol) compared to LB alone, and this in turn results in robust biofilm formation [8]. Moreover, glycerol and Mn^2+^ may induce a network of gene expression leading to purine biosynthesis, GTP homeostasis and nucleotide signaling that transforms low-biofilm-forming LB into a robust biofilm-inducing medium [8]. It is well established that Mn^2+^ promotes biofilm formation by *B*. *subtilis* in several growth media [7,16].

Here, we established that the promotion of *B*. *cereus* biofilm formation by Mn^2+^ is strictly dependent on the strain tested. Moreover, we showed that the role of glycerol in Mn^2+^-dependent induction of *B*. *cereus* biofilm formation is very minor (Fig 1). The addition of glucose and Mn^2+^ to BHI (BHI+Glc+Mn) resulted in less biofilm formation than the addition of Mn^2+^ alone. These results indicate that Mn^2+^-related induction of *B*. *cereus* biofilm formation through the KinD system is independent of the presence of excess glycerol or glucose in BHI medium. These results are consistent with the results of a previous study by Gao el al. [6] in which it was shown that addition of excess glucose and glycerol to BHI does not promote biofilm formation in *B*. *cereus*. Moreover, although the addition of glucose to tryptic soy broth (TSB) promotes biofilm formation, the addition of glycerol to TSB reduces biofilm formation. However, these data are inconsistent with the results of previous studies by Shemesh et al. [7] and Yan et al. [8], who reported that a combination of glycerol and Mn^2+^ in LB medium only promotes biofilm formation in *B*. *cereus*. These conflicting results might be related to the different compositions of BHI and LB media; the former contains glucose (2 g/L), whereas LB lacks glucose. The idea that the composition of LB might play a major role in biofilm formation is also suggested by the study of Mhatre et al. [16], which showed that Mn^2+^ related induction not only depends on the addition of glycerol but that addition of glucose to LB can also induce higher biofilm formation.

Our data show that supplementation of BHI medium with a combination of Mn^2+^ and heme can transform BHI into a medium that supports robust biofilm formation by several *B*. *cereus* strains. However, the addition of heme alone to BHI had no effect on biofilm formation by *B*. *cereus*. The study of Hayrapetyan et al. [5] demonstrated that *B*. *cereus* can utilize heme (hemin) as an iron source but that this ability is highly strain-specific. Furthermore, the combination of Mn^2+^ and heme in BHI might also play an important role in Mn^2+^ and heme intake and cellular metabolism utilization proficiency. Previously, it has been shown that the ratio of Mn^2+^ to heme plays an important role in spore resistance in *B*. *subtilis* [30].

We performed cell enumeration to determine the impact of the addition of Mn^2+^ and heme to BHI on the number of cells within biofilm complexes. Our results revealed a good correlation between the results of crystal violet assays and the number of cells present, in agreement with our recent study of *B*. *cereus* biofilms [31]. In addition, the results of crystal violet assays and enumeration of the number of cells were consistent with the results obtained by SEM image analysis. Moreover, the viability of GIHE 72–5 cells within biofilm matrix on plastic coupons tested using LIVE/DEAD staining showed results consistent with the crystal violet assays, cell enumeration experiments and SEM images. The fact that there were no significant (p > 0.05) in the percentage of dead cells under addition of Mn^2+^ or heme, in particular heme, indicated that heme did not result in cell lysis due to heme-related toxicity (S1 Table).

Here, we showed that addition of Mn^2+^ to BHI significantly increased sporulation efficacy in *B*. *cereus* compared to BHI alone. Previous studies have shown that addition of Mn^2+^ to the culture medium induces sporulation in *Bacillus* biofilms [16]. In the planktonic growth phase of *Bacillus*, a similar induction of sporulation in several growth media after the addition of Mn^2+^ has been reported [32–34]. However, Mn^2+^-dependent sporulation in *Bacillus* requires the presence of an additional carbon source such as glucose or glycerol [32]. Transcriptome analysis in *B*. *subtilis* shows that Mn^2+^-dependent induction is related to the expression of spore core proteins (i.e., gerPB, gerPD and gerPE), which are downregulated in the absence of Mn^2+^ [16]. Our results revealed that a combination of Mn^2+^ and/or heme in BHI was favorable for high sporulation in *B*. *cereus* biofilms on SS coupons, although heme alone had no effect. Similar results have been reported for *B*. *subtilis* planktonic cells, in which iron has been shown to induce sporulation in several growth media [34]. Moreover, sporulation efficacy depends on the form in which iron is supplied; soluble iron salts are more active than insoluble iron in inducing sporulation [34]. To date, there have been no studies of the effects of supplementation of BHI with a combination of Mn^2+^ and heme on sporulation efficiency in *Bacillus*. However, it was previously demonstrated that although Mn^2+^ is not essential for the resistance properties of *B*. *subtilis* spores, the ratio of Mn^2+^ and iron is important in determining spore resistance to disinfectants (ionizing radiation) [30].

The data on bacterial motility showed that addition of Mn^2+^ to BHI was positively correlated with swarming ability and a robust biofilm formation phenotype. This result is consistent with the results of previous studies in which it was shown that the swarming ability of *B*. *cereus* plays a major role in the initial attachment of the bacteria to a suitable surface and in subsequent biofilm formation [20,35,36]. A deletion mutation of clpYQ in *B*. *cereus* AR 156 showed reduced swarming zone size and low biofilm formation compared to wild type in LB medium supplemented with Mn^2+^ and glycerol (LBGM), suggesting a role of clpYQ in swarming ability and biofilm formation [35]. Interestingly, here we found that addition of heme to BHI also increased swarming ability; however, heme addition had no major impact on biofilm formation (Figs 1 and 4). These data indicate that *B*. *cereus* biofilm formation is not only dependent on swarming ability but is a complex process that involves several pathways [5,8].

The role of Mn^2+^ and heme in the motility of *B*. *cereus* grown in BHI and in subsequent robust biofilm formation was further established based on the results obtained regarding the expression of a number of selected genes. After addition of Mn^2+^ to BHI, in almost all conditions, *spo0A*, *sinI*, *sipW* and *tasA* were expressed at several-fold higher levels under biofilm growth phase conditions compared to its expression during growth in BHI alone (Fig 7C). These results indicate that *spo0A-sinI-sinR* and *sipW-tasA* regulatory pathways play an important role in the Mn^2+^-regulated induction of *B*. *cereus* biofilm formation. The higher expression of the *spo0A* and *sinI* as well as lower expression of *sinR* genes during *B*. *cereus* biofilm formation is consistent with the results of previous studies [6,20]. Recently, Xu et al. [20] reported that the *spo0A*-*sinI*-*sinR* expression pathway plays the key regulatory role during biofilm formation by *B*. *cereus*. In addition, deletion of *sp0A* and *sinI* genes were shown to result in defects in swarming ability [23] and pellicle formation in *B*. *cereus* [6]. Moreover, the higher expression of *sipW* and *tasA* during addition of Gly, Mn and heme indicate that these compounds promote *B*. *cereus* biofilm formation through *sipW-tasA* genetic pathway. The expression of TasA protein that forms the amyloid-like fibers in response to Gly, Mn and heme in BHI was also clearly visible in swarming experiment photos (Fig 5). These results further revealed that the robust biofilm formation in response to Gly, heme and Mn of *B*. *cereus* might be mediated through the *sipW-tasA* regulatory pathway. In contrast, here we found that *abrB* gene expression was downregulated under almost all conditions during the biofilm growth phase, whereas in the planktonic growth phase the addition of Mn^2+^ to BHI resulted in several-fold higher expression of the gene compared to BHI alone. These data indicate that *abrB*, a central regulatory gene that plays a key role in biofilm formation in *B*. *subtilis* by regulating cell mobility and differentiation [23], plays a major role in the response of *B*. *cereus* to the addition of Mn^2+^ to BHI during planktonic growth conditions. At the early stage of biofilm formation, planktonic cells require a suitable surface for attachment; *abrB* gene expression is upregulated at this stage, leading to increased expression of a network of motility-related genes such as *lytA* and *lytF*. However, in mature biofilms, *abrB* expression is downregulated, which turns on the expression of matrix genes (*eps*, *tapA*, *bslA*) and turns off the expression of motility-related genes [23]. The downregulation of *abrB* gene expression after the addition of Mn^2+^ to BHI observed in our study suggests that motility plays a key role in inducing *B*. *cereus* biofilm formation at the early stage. Our results showed a clear correlation between BAC resistance and robust biofilm formation in *B*. *cereus* grown in BHI containing Mn^2+^ and heme. The data suggest that robust biofilm formation by *B*. *cereus* grown in BHI in the presence of Mn^2+^ and heme may be due to high biomass formation (indicated by the results of the crystal violet assay), the formation of dense cell clusters, and sporulation under these growth conditions.

In conclusion, in this study we showed that addition of Mn^2+^ and heme to BHI induced robust biofilm formation by *B*. *cereus* and that this occurred in a strictly strain-dependent manner. Biofilm formation phenotypes such as cell density, sporulation, biofilm architecture and resistance to disinfectant were largely affected by addition of Mn^2+^ and heme to BHI. The pattern of expression of a number of selected genes suggest the involvement of Mn^2+^ and heme in the motility and biofilm formation of *B*. *cereus* through *sp0A-sinI-sinR* and *sipW-tasA* regulatory pathways. The results of this study indicate that *B*. *cereus* biofilm formation can be altered in the presence of signaling molecules such as Mn^2+^ and heme. Because these molecules are widely available in the food-processing environment, this could have a significant detrimental effect on the food industry.

## Supporting information

S1 Fig Impact of addition of glucose to Mn^*2+*^ and heme on biofilm formation by *B. cereus* food isolate GIHE 72–5. Biofilms were grown on SS coupons with or without Mn^2+^ and/or heme in BHI supplemented with glucose at 30°C for 48 h. Established biofilms were quantified using the crystal violet assay. The threshold of biofilm formation (solid line), is equal to the background absorbance value plus three times the standard deviation (OD = 0.3). Each data point represents the average value obtained in three biological experiments with each strain; the error bars indicate standard deviation. To compare effects on biofilm formation in each strain, one-way ANOVA and Tukey's post hoc test (p < 0.05) were performed. Groups marked with different letters in each strain display significant differences.
(TIF)

S2 Fig Impact of Mn^2+^ and heme on planktonic grown cultures of *B. cereus*. *B*. *cereus* GIHE 72–5 planktonic cells were grown in BHI supplemented with glycerol with or without Mn^2+^ and/or heme at 30°C for 48 h. Number of cells in logCFU/ml (A); scatter plot showing the relationship between the number of cells and the results of crystal violet assays for biofilm formation (B). Each data point represents the average value obtained in three biological experiments, and the standard deviation. To compare the number of cells and spores obtained among the conditions used, one-way ANOVA and Tukey's post hoc test (p < 0.05) were performed. Groups marked with different letters in each growth condition display significant differences.
(TIF)

S3 Fig Impact of Mn^2+^ and heme on biofilm formation by the *B. cereus* food isolate GIHE 72–5 on plastic coupons. Biofilms were grown with or without Mn^2+^ and/or heme in BHI supplemented with glycerol at 30°C for 48 h. Established biofilms were quantified using the crystal violet assay (A) and cell enumeration (B). The threshold of biofilm formation (solid line) is equal to the background absorbance value plus three times the standard deviation (OD = 0.3). Each data point represents the average value obtained in two biological experiments for each strain; error bars indicate the standard deviations. To compare effects on biofilm formation in each strain, one-way ANOVA and Tukey's post hoc test (p < 0.05) were performed. Groups marked with different letters in each strain display significant differences.
(TIF)

S4 Fig Impact of Mn^2+^ and heme on the expression of a number of selected genes in *B. cereus* in the planktonic growth phases. The graph shows the expression of *spoOA* (A), *abrB* (B), *sinI* (C), *sinR* (D), *sipW* (E) and *tasA* (F) in the planktonic growth phase of GIHE 72–5. Planktonic cells were grown in Mn^2*+*^ and/or heme in BHI supplemented with glycerol at 30°C for 48 h. The fold change in expression relative to the expression in planktonic cells grown in BHI is shown. Each data point represents the average value obtained in at least two biological experiments; the error bars indicate standard deviation. To compare fold changes in expression under different conditions, one-way ANOVA and Tukey's post hoc test were performed. Groups marked with different letters in each growth condition display significant differences (p < 0.05).
(TIF)

S1 Table Impact of Mn^2+^ and heme on viability of cells within *B. cereus* GIHE 72–5 biofilms. * the percentage of live and dead cells were determined according to previously published protocols [1]. Groups marked with different letters in each column show significant differences (one-way ANOVA and Tukey's post hoc test, p < 0.05).
(DOCX)
