# Supplementary material for: Impact of manganese and heme on biofilm formation of Bacillus cereus food isolates
Source: PLoS One. 2018 Jul 26;13(7):e0200958. doi: 10.1371/journal.pone.0200958 (PMC6062052; doi:10.1371/journal.pone.0200958)
Supplement: S1 Table — * the percentage of live and dead cells were determined according to previously published protocols [1]. Groups marked with different letters in each column show significant differences (one-way ANOVA and Tukey's post hoc test, p < 0.05). (DOCX) [file pone.0200958.s005.docx]

**S1 Table.**

| *B. cereus* strain | Live cells (%)* | Dead cells (%)* |
| --- | --- | --- |
| BHI | 68 ± 2^c^ | 32 ± 2^c^ |
| BHI+Gly | 68 ± 1^c^ | 32 ± 1^c^ |
| BHI+Mn | 53 ± 1^a^ | 47 ± 1^e^ |
| BHI+Heme | 88 ± 3^d^ | 12 ± 3^a^ |
| BHI+Mn+Heme | 80 ± 2^d^ | 20 ± 2^b^ |
| BHI+Gly+Mn | 80 ± 1^d^ | 20 ± 1^b^ |
| BHI+Gly+Heme | 62 ± 3^bc^ | 36 ± 1^cd^ |
| BHI+Gly+Mn+Heme | 60 ± 0^ab^ | 40 ± 0^d^ |

# References

1. Chávez De Paz LE. Image Analysis Software Based on Color Segmentation for Characterization of Viability and Physiological Activity of Biofilms. Appl Environ Microbiol. 2009;75: 1734–1739. doi:10.1128/AEM.02000-08
